# (1*S*,2*S*)-Cyclohexane-1,2-diamine-based Organosilane Fibres as a Powerful Tool Against Pathogenic Bacteria

**DOI:** 10.3390/polym12010206

**Published:** 2020-01-14

**Authors:** Veronika Máková, Barbora Holubová, David Tetour, Jiří Brus, Michal Řezanka, Miroslava Rysová, Jana Hodačová

**Affiliations:** 1 Department of Nanomaterials in Natural Science, Institute for Nanomaterials, Advanced Technologies and Innovation, Technical University of Liberec, Studentská 1402/2, 461 17 Liberec, Czech Republic; barbora.holubova@tul.cz (B.H.); michal.rezanka@tul.cz (M.Ř.); 2 Department of Organic Chemistry, University of Chemistry and Technology, Prague, Technická 5, 166 28 Prague, Czech Republic; davidtetour@seznam.cz (D.T.); jana.hodacova@vscht.cz (J.H.); 3 Institute of Macromolecular Chemistry, Academy of Sciences of the Czech Republic, Heyrovsky Sq. 2, 162 06 Prague, Czech Republic; brus@imc.cas.cz; 4 Department of Nanomaterials and Informatics, Institute for Nanomaterials, Advanced Technologies and Innovation, Technical University of Liberec, Studentská 1402/2, 461 17 Liberec, Czech Republic; miroslava.rysova@tul.cz

**Keywords:** organic-inorganic hybrids, organo-bridged silsesquioxane, sol-gel process, electrospinning, *Staphylococcus aureus*, *Pseudomonas aeruginosa*

## Abstract

An urgent need to find an effective solution to bacterial resistance is pushing worldwide research for highly effective means against this threat. Newly prepared hybrid organosilane fibres consisting of a (1*S*,2*S*)-cyclohexane-1,2-diamine derivative, interconnected in the fibre network via covalent bonds, were fully characterised via different techniques, including FTIR, TGA-FTIR, SEM-EDS, and solid-state NMR. Fibrous samples were successfully tested against two types of pathogenic bacterial strains, namely *Staphylococcus aureus*, and *Pseudomonas aeruginosa*. The obtained results, showing >99.9% inhibition against *Staphylococcus aureus* and *Pseudomonas aeruginosa* in direct contact compared to the control, may help particularly in case of infections, where there is an urgent need to treat the infection in direct contact. From this point of view, the above-mentioned fibrous material may find application in wound healing. Moreover, this new material has a positive impact on fibroblasts viability.

## 1. Introduction

Pathogenic bacteria have become a worldwide problem. According to the World Health Organization (WHO) statistics, over 1.4 million people worldwide suffer from infections caused by pathogenic bacteria acquired in hospitals [1]. *Staphylococcus aureus* (*S. aureus*) and *Pseudomonas aeruginosa* (*P. aeruginosa*) are among the most problematic pathogenic bacteria closely connected with an extremely overgrowing resistance to antibiotics, which brings another major complication in the treatment [2,3]. The infections caused by *P. aeruginosa* are usually resistant to multiple antibiotics due to the bacterium’s intrinsic resistance [4]. It is important to note that both bacterial strains are 1000 times more resistant in the form of a biofilm [5].

Due to the antibiotic resistance of these bacteria, great effort has been devoted to developing new antibiotics. One group of such compounds are, for example, *trans*-cyclohexane-1,2-diamine derivatives (DACHs). Previous studies have proved them to be very promising antibacterial compounds against *P. aeruginosa* [6], Mycobacterium tuberculosis [7,8,9], and *S. aureus* [6,8]. Moreover, it was found that (1*S*,2*S*) enantiomers generally have lower minimum inhibitory concentrations compared to racemate [6].

Functional organosilica or organosilane-based hybrids for biomedicine applications are well known to the academic public and in various industrial applications [10,11,12,13,14]. In general, an ever-growing interest [13] lies not only in the mild synthesis conditions offered by the sol-gel process producing a broad range of end-products but also in the possibility of nanoscale tailoring of their chemical structure fulfilling the specific needs of biocompatibility and mechanical properties of targeted bio-applications [10,11,12,13,14,15,16].

Organosilica and organosilane materials with antibacterial properties are found particularly in the form of nanoparticles [17] or thin coatings [18] on various substrates. Surprisingly, the preparation of the hybrid fibres based on bridged organo-bis-silylated precursors is rarely reported [19,20]. Furthermore, in most cases, hybrid fibres are produced as a combination of silica alkoxides or organo-mono-silylated precursors mixed with organic polymers (poly(ε-caprolactone)/polyethylene terephthalate/polyvinylalcohol etc.) or biopolymers [16,18,21,22]. However, such materials are less degradable, and often inappropriate solvents, or precursors are employed. An example of such material is polyhydroxybutyrate/poly(ε-caprolactone)/silica hybrid nanofibrous scaffolds for bone tissue regeneration [23,24] or electrospun organosilane-loaded collagen nanofibrous scaffolds containing quaternary ammonium organosilane and octadecyldimethyl(3-trimethoxysilylpropyl)ammonium chloride with antibacterial activity [25].

To the best of our knowledge, a fibrous system combining the advantages of organosilane and cyclohexane-1,2-diamine derivatives has not been studied yet (or even prepared). This system may offer an answer to the urgent need for the development of new types of biomaterials, which may help to improve the problem of difficult-to-heal infections (wounds) caused by pathogenic bacteria in direct contact. This project deals with challenges in the preparation of antibacterial fibres with a pure organosilane composition based on a silylated (1*S*,2*S*)-cyclohexane-1,2-diamine precursor. No surfactants, low-molecular-weight polymeric gelators, or spinnable polymers were used during the subsequent electrospinning process, and the whole one-pot synthesis is based on a one solvent solution to minimise the toxicity of the formation of the fibres and their subsequent use.

## 2. Materials and Methods

Tetraethyl orthosilicate—TEOS (>99%, Merck, Prague Czech Republic), ethanol—EtOH (99.9%, Penta, Prague, Czech Republic), hydrochloric acid (35%, Prague, Czech Republic), deionised water (Milli-Q, Prague, Czech Republic). Aluminium foil thickness 12 µm (Rotilabo, Karlsruhe, Germany). (1*S*,2*S*)-1,2-Bis{*N*’-[3-(triethoxysilyl)propyl]ureido}cyclohexane (compound 1) was prepared according to the literature [26].

The standard procedure for the synthesis of a spinnable organosilane sol solution was as follows: Silica precursor composed of TEOS:compound 1 (in a molar ratio of 57:43), ethanol, deionised water, and HCl were added into a round bottom flask and stirred at 450 rpm/r.t./30 min (Table 1). The pH of the sols was adjusted to 2. Finally, the solution was heated to 90 °C in an oil bath at 450 rpm, refluxed for 4 h, and subsequently partially evaporated. The dynamic viscosity was measured before the electrospinning and appropriately adjusted. Hybrid fibres (marked as DACHsilane) were prepared via jet needle electrospinning (Table 1) and finally dried for 24 h in a desiccator for further use.

Inorganic silica dioxide nanofibres with an average diameter of 0.33 ± 0.08 μm, prepared according to the already-published procedure [27], were used as a standard material to assess antibacterial and cytotoxicity activity.

### 2.1. Characterisation Techniques

#### 2.1.1. Scanning Electron Microscopy

The morphology of the hybrid DACHsilane fibres was studied by SEM (ZEISS Ultra Plus, Sigma Family, Jena, Germany). Samples were sputtered with a 2-nm platinum layer and were subsequently viewed as secondary electron images (1 kV). The fibre diameter was characterised using the NIS Elements software (LIM s.r.o., Liberec, Czech Republic) and was assessed from a total amount of 100 measurements per material, taken from five independent images. The results were evaluated in the form of the mean ± standard deviation.

Energy-dispersive X-ray spectroscopy (SEM-EDS, Oxford X-MAX 20, ZEISS Ultra Plus, Sigma Family, Jena, Germany) was used at 10 keV to evaluate the chemical composition of the prepared fibrous (DACHsilane) samples.

#### 2.1.2. Fourier Transform Infrared Spectroscopy (FTIR)

FTIR spectrometry was used to assess the chemical structure of the prepared hybrid DACHsilane fibres. FTIR Spectrometer Nicolet iZ10 (Thermo Fisher Scientific, Waltham, MA, USA) with an attenuated total reflection (ATR) diamond crystal angle of 45°, and a spectral range of 4000–700 cm^−1^ was used for the analysis. The number of sample scans: 16, number of background scans: 32, resolution: 4 cm^−1^, gain: 4.0, apodisation: Happ–Genzel, correction: Atmospheric suppression, baseline.

#### 2.1.3. ^29^Si cross Polarization Magic Angle Spinning Nuclear Magnetic Resonance (CP/MAS NMR) Spectroscopy

Solid state NMR analysis was used to evaluate the chemical structure of the prepared DACHsilane fibres. Spectra were measured at 11.7 T using a Bruker AVANCE III HD WB/US NMR spectrometer (Bruker, Ettlingen, Germany) in a double-resonance 4-mm probe head at spinning frequencies ω_r_/2π = 7 and 10 kHz. In all cases, finely powdered dry samples were placed into 4-mm ZrO_2_ rotors. All of the experiments were conducted at 303 K. The ^29^Si CP/MAS NMR spectra were recorded at Magic Angle Spinning (MAS) of 7 kHz. The spectra were referenced to the external standard M8Q8 (−109.8 ppm). The number of scans was 0.5–5k depending on the amount of sample. For the quantitative analysis and deconvolution of ^29^Si CP/MAS NMR spectra, the TopSpin 3.6 program package (Bruker) was used [28].

#### 2.1.4. Thermogravimetric Analysis Fourier Transform Infrared Spectroscopy (TGA-FTIR)

The samples were analysed using a Q500 thermogravimetric analyser (TA Instruments, New Castle, PA, USA). Each sample was placed on a platinum pan and analysed in a non-reactive atmosphere of nitrogen with a flow rate of 60 mL/min. The samples were heated at 10 °C/min; the range was from 20 °C to 650 °C. The thermal decompose products were analysed by the Nicolet iS10 FTIR spectrometer (Thermo Fisher Scientific, Waltham, MA USA). Spectra were taken every 10 s with a resolution of 2 cm^−1^ in the spectral range of 4000–650 cm^−1^.

### 2.2. Antibacterial Activity Assessments

All of the tested fibrous samples (round shape, the diameter 0.6 cm) were sterilised for 1 h at 120 °C before each bacterial experiment separately. Pure silica dioxide (SiO_2_) fibres were used as standard comparison material in all experiments.

Antibacterial tests were performed using Gram-positive *Staphylococcus aureus* (CCM 3953) and Gram-negative *Pseudomonas aeruginosa* (CCM 3955) (ALE-G18, CSNI, collection of microorganisms, Masaryk University, Brno, Czech Republic). A Luria−Bertani (LB) broth medium was used to prepare the agar plates (Sigma-Aldrich, Merck, Czech Republic).

#### 2.2.1. Qualitative Method

The Kirby–Bauer test was used to analyse the ability of both types of fibres (pure inorganic SiO_2_ and hybrid DACHsilane) to inhibit the growth of *S. aureus* and *P. aeruginosa*. A total of 1 mL of both the bacterial inocula (initial optical cell density at 600 nm 0.15 ± 0.08 (McFarland standard concentration = 0.9 × 10^7^ CFU/mL)), was spread over four LB agar plates separately using sterile swabs. The samples (round shape, the diameter 0.6 cm) were placed in the centre of the agar plate and incubated for 24 h at 37 °C. The bacterial growth-inhibiting effect was determined by the size of the inhibition zone around the samples.

#### 2.2.2. Quantitative Method

The colony-forming unit (CFU) counting method was used to evaluate the antibacterial activities of the pure inorganic SiO_2_ fibres (used as a standard comparison material) and newly prepared hybrid DACHsilane fibres against *S. aureus* and *P. aeruginosa* following the reported protocol [29].

The overnight cultures of bacteria (50 mL) in the Luria−Bertani (LB) broth were centrifuged for 5 min at 3780 rcf (relative centrifugal force) to remove supernatant, washed with hosphate-buffered saline (PBS) twice and then re-suspended in a sterile physiological saline solution (0.15 M NaCl, pH 7.0, 20 mM NaHCO_3_) to an initial optical cell density at 600 nm (OD600) of 0.15 ± 0.08 (McFarland standard concentration = 0.9 × 10^7^ CFU/mL)).

Fibrous samples with an approximate diameter of 0.6 mm were placed in sterile Fisher bottles (100 mL) with 20 mL of the prepared bacteria solution separately and were incubated for 5 h at 37 °C. Subsequently, the samples were taken out of the bacteria solution, put into sterile glass vials with 10 mL of physiological saline solution, and were gently shaken in a shaker (Heidolph Unimax 1010, Thermo Fisher Scientific, Pardubice, Czech Republic) for 7 min at 25 °C to remove the attached bacteria. The obtained suspensions were diluted 100 times, and 100 μL of each bacterial solution was taken to plate on (LB) agar plates. Each test was performed in triplicate. Viable colonies of microbes on the agar plate were counted, and the percentage of cell growth reduction (CGR, %) was calculated using the equation CGR = (C_0_ − C/C_0_) × 100%, where C_0_ is the number of CFU of bacteria from the control sample and C is the number of CFU of bacteria from hybrid DACHsilane fibres [1,29].

#### 2.2.3. Bacterial Adhesion

The bacterial adhesion of *S. aureus* and *P. aeruginosa* on the surface of each fibrous sample was evaluated according to the reported literature [29]. Several changes, appearing during the experiment, are mentioned below. The prepared samples were incubated in the bacterial solutions at the initial optical cell density (0.15 ± 0.08/600 nm) for 1 h at 37 °C. Subsequently, the samples were rinsed twice with distilled water, put on glass slides and covered with a Live/Dead Backlight, 1 Kit 30× diluted solution containing (1.67 mM of SYTO9—A and 18.3 mM propidium iodide—B, molar ratio 1:1). The samples were kept in the dark for 15 min and further analysed at 630 nm using the filters 44 fluorescein isothiocyanate (FITC) (green) and 43 cy3 (red). The live and dead bacteria (*S. aureus* and *P. aeruginosa*) attached to the surface of the samples were imaged using a fluorescent microscope (ZEISS Axio Imager 2, Jena, Germany).

### 2.3. Cytotoxicity Experiments

Cytocompatibility of the DACHsilane hybrid fibres was assessed using murine fibroblasts 3T3-A31. Prior to testing, the cells were maintained in a completed DMEM medium (4.5 g/L d-glucose, l-glutamine, sodium pyruvate, Sigma-Aldrich-Merck, CZ) supplemented with 5% foetal bovine serum (FBS, Biosera, CZ), 5% new-born calf serum (NBCS, Sigma-Aldrich-Merck, CZ), and a 1% penicillin-streptomycin (PS) antibiotic mixture. The cell viability assay was performed in compliance with the ISO 10993-5:2009 standard with minor modifications. Briefly, the 3T3-A31 fibroblasts were seeded into a 96-well plate (10,000 cells per well) and cultured for 24 h prior to the main experiment. The UV-C sterilised samples were extracted in a supplement-free media for 24 h (37 °C/100 rpm). After the extraction period, macroscopic residues were removed by sterile filtration. The obtained filtrates were supplemented by 5% FBS/5% NBCS/1% PS to final concentrations corresponding to 500 µg, 250 µg, and 125 µg fibres per 1 mL of complete medium. The cells were exposed to 100 µL of these extracts per well for 24 h under standard conditions (37 °C/5% CO_2_). Each sample was tested six times and compared to the cell control (pristine complete medium), positive control (0.1% Triton x-100), and negative control (0.01% l-arginine). The inorganic silica dioxide nanofibres were used as a secondary control and went through the same elution process described above. After exposure for 24 h, the 3-(4,5-dimethyl-2-thiazolyl)-2,5-diphenyl-2*H*-tetrazolium bromide (MTT, Sigma-Aldrich-Merck, Prague, Czech Republic) viability assay was performed and evaluated by an absorbance reading at 570 nm. The final viability was calculated as a proportion of the cell control value. The results of the cell viability assay were supported by cell morphology evaluation, as described in Figure S6 in the Supplement.

## 3. Results and Discussion

**Figure 1:**
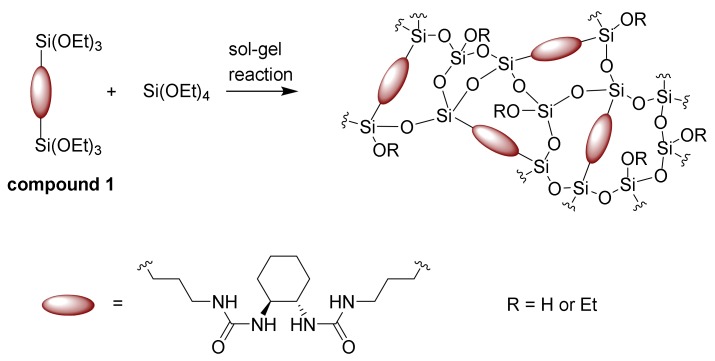
The sol-gel process of forming pure hybrid organosilane fibres based on compound **1**.

A novel hybrid fibrous material marked as DACHsilane was prepared via electrospinning (Figure 1). Bis-silane precursors are known to behave as network builders during polymerisation and, hence, the resulting fibrous structures display unique material properties. The incorporation of compound **1** then leads to the formation of a hybrid material. This results in a rather weakly-branched, extended polysiloxane matrix required for the fibre-making process [20,30,31,32].

### 3.1. Characterisation of Pure Organosilane Fibres with DACH Functionality

**Figure 2:**
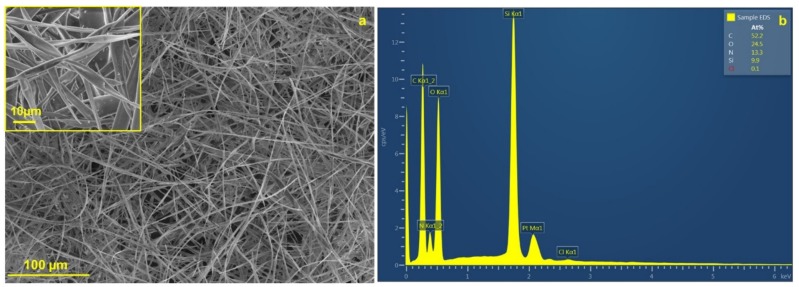
SEM image of the prepared hybrid fibres with an inset showing the helical structure of the parts of the fibres (**a**); Energy Dispersive X-Ray Spectroscopy (EDS) spectra of the hybrid fibres (**b**).

Morphologically compact and homogeneous fibres with a diameter of 2.47 ± 0.91 μm (Figure 2a) were obtained via the needle electrospinning method. The prepared organosilane fibres are flexible and do not break during sample manipulation. Some of the fibres have a tendency to form in the helical structure, probably during the electrospinning procedure (inset image in Figure 2a).

**Figure 3:**
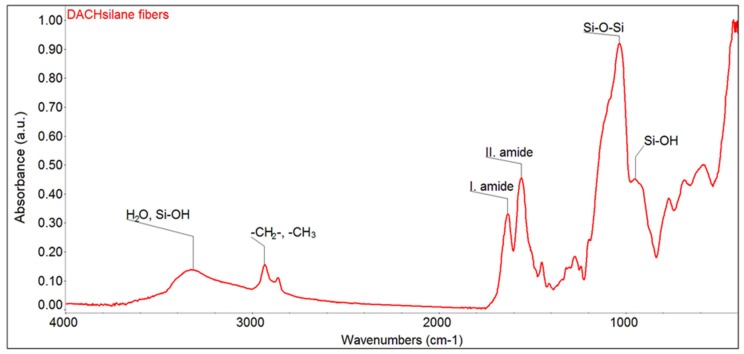
FTIR spectra of the prepared hybrid DACHsilane fibres made of compound **1**.

EDS analysis confirmed the presence of oxygen, carbon, nitrogen, and silicon in the fibrous structure (see Figure 2b). The visibility of nitrogen in the spectra is attributed to the urea functional groups. Moreover, this observation is in compliance with the FTIR result (Figure 3), which confirms the appearance of the characteristic vibrations corresponding to the urea NH-CO-NH and cyclic aliphatic hydrocarbon functionalities in the fingerprint area from 1700 to 1200 cm^−1^ (various stretching and bending vibrations). Primarily, the doublet at around 1630 and 1560 cm^−1^ (urea units; ν(CO) and δ(NH)) and the less intense peak at 1450 cm^−1^ (cyclic aliphatic hydrocarbon unit, δ(CH_2_)) provide strong evidence [33,34,35] that compound **1** is successfully preserved in the prepared organosilane fibrous material. Moreover, the area from 1200 to 400 cm^−1^ proves that no Si–C cleavage occurred in greater amounts, and the silica units progressed well towards the interconnected polysiloxane Si–O–Si network [33,36].

**Figure 4:**
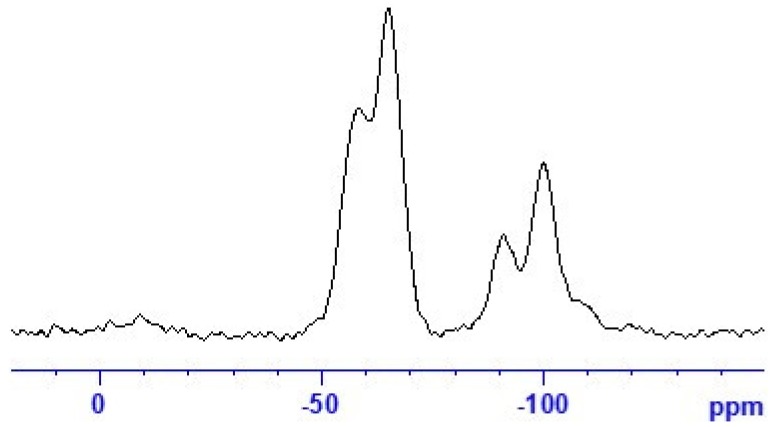
^29^Si CP/MAS NMR spectra of the hybrid DACHsilane fibres.

Spectroscopic ^29^Si CP/MAS NMR examinations further confirm the correct setting of sol-gel processing parameters in accordance with the above-mentioned research on the sol-gel processing of silica fibres [20,31,32]. The recorded ^29^Si CP/MAS NMR spectrum of the studied organosilane mat (Figure 4) exhibits five clearly resolved resonances corresponding to the characteristic structure units of the siloxane network at different condensation reaction rates: two well-pronounced T*^n^* signals (the organosilane bridged precursor; C–Si(OSi)*_n_*(OH)_3-*n*_) and three Q*^n^* signals (the polysiloxane TEOS matrix; Si(OSi)*_n_*(OH)_4-*n*_) [31,33,34]. The overall composition of the siloxane fractions (Table 2) is dominated by incompletely condensed T^2^, Q^2^, and Q^3^ species together with a completely polymerised T^3^ unit. These findings most probably indicate the formation of a ladder-like structure leading to a spinnable linear polymer (Figure 1), as fully-grown 3D Q^4^ species would be rather unsuitable for electrospinning. As the organosilane (**1**) silicon prevails (compound **1**:tetraethoxysilane (TEOS) = 43:57 mol.%) in the composition formula, the T*^n^* signals also dominate in the spectrum. The effect of the hydrolysis-polycondensation reaction inevitably leads to the copolymerisation between the structural units of TEOS and the organosilane modifier [31,37]. Hence, the successful formation of a Class II hybrid material [10] may be confirmed.

The thermogravimetric analysis coupled with FTIR spectroscopy (Figure S1) indicates that the prepared fibrous organosilane material is stable up to 200 °C, where a major weight loss begins and reaches a maximum at around 260 °C. This loss is attributed to the decomposition of the urea unit within the hybrid organosilane network. The TGA-FTIR analysis also confirms the presence of a small portion of water or solvent contained in the fibrous mat most probably originating from incomplete hydrolysis-polycondensation, as previously indicated in the ^29^Si CP/MAS NMR spectroscopic evaluation.

### 3.2. Biomedical Applications

#### 3.2.1. Assessment of the Inhibition Zone—Antibacterial Activity in Direct Contact

A qualitative test was performed to evaluate the antibacterial activity of the prepared hybrid DACHsilane fibres in direct contact (Figure S2). The prepared hybrid fibres exhibited a significant antibacterial effect, particularly against *S. aureus* in direct contact (Figure S2b in detail), rather than in the case of *P. aeruginosa*. Standard material, pure SiO_2_ fibres, showed no antibacterial activity in direct contact for both of the tested bacterial strains after 24 h incubation (Figure S2a,c). Moreover, several bacterial colonies were observed directly under the standard samples in direct contact. All of the Petri dishes were densely populated by the bacterial colonies, except for very small areas around or under the samples made of hybrid DACHsilane fibres. The inhibition of bacteria for both strains was observed below the hybrid DACHsilane fibres (inset images Figure S2b,d) Moreover, the DACHsilane fibres showed a halo zone hint around the sample in the case of *S. aureus* (Figure S2b).

#### 3.2.2. Assessment of Antibacterial Activity

**Figure 5:**
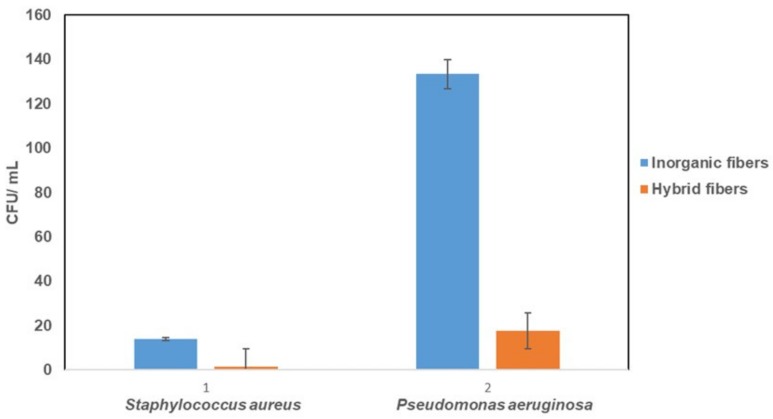
Comparison of the antibacterial activity for pure inorganic SiO_2_ fibres and hybrid DACHsilane fibres against *S. aureus* and *P. aeruginosa*.

The results related to the antibacterial activity in the solution were examined by calculating the bacterial cell growth reduction (CGR %) using Gram-positive bacteria *S. aureus* and Gram-negative bacteria *P. aeruginosa*. Figure 5 shows visible differences between the purely inorganic and hybrid DACHsilane fibres. The examined hybrid fibrous samples seemed to show significant antibacterial activity compared to the control and standard inorganic fibrous samples (Table S1, Figures S3–S5 in the Supplement). The antibacterial activity of the purely SiO_2_ fibrous material was significantly less compared to the tested hybrid fibrous material. Better results were observed against *S. aureus* with 91% antibacterial activity than against *P. aeruginosa,* where the antibacterial activity reached only 87%. When we compare these results to the control for cell bacterial growth (Figure S3), the inhibition activity for both bacterial strains is even higher than 99.9%, which is in close agreement with the recent literature [25]. The results obtained from this experiment (Figure 5) closely correspond to the bacterial adhesion test mentioned below, where *P. aeruginosa* has a higher tendency to adhere to the surface of the hybrid fibrous sample compared to *S. aureus*.

#### 3.2.3. Bacterial Adhesion Activity

**Figure 6:**
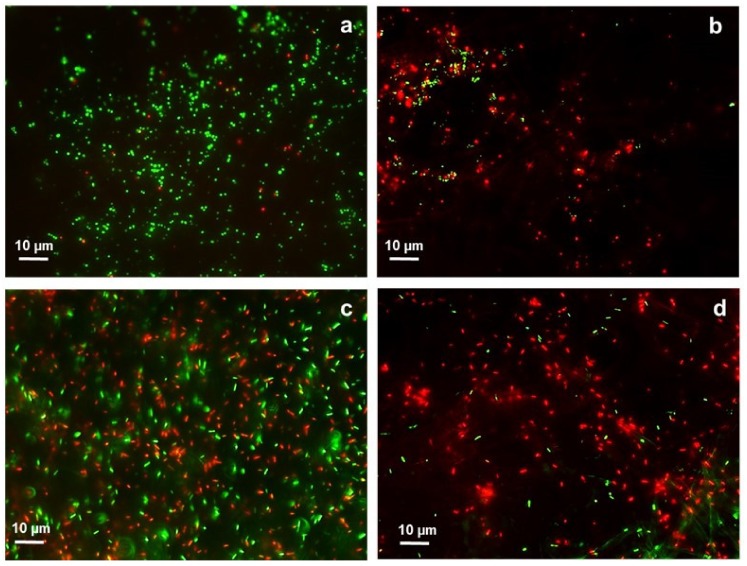
Fluorescence microscopy images of *S. aureus* on the surface of inorganic SiO_2_ fibres (**a**) and on the surface of hybrid DACHsilane fibres (**b**). *P. aeruginosa* present on the surface of inorganic SiO_2_ fibres (**c**) and on the surface of hybrid DACHsilane fibres (**d**). Green dots indicate live cells, while red dots dead cells.

The bacterial adhesion to the surfaces of the fibrous samples against *S. aureus* and *P. aeruginosa*, was assessed by fluorescence imaging, see Figure 6. The green and red fluorescent spots indicate the live and dead bacteria, respectively. They were observed on the surface of the inorganic and hybrid fibres after 1 h incubation in the bacterial suspensions. The inorganic fibrous samples mostly exhibited green fluorescent spots scattered on the surface in both cases, suggesting live *S. aureus* and *P. aeruginosa* (Figure 6a,c). This fact indicates a lack of antibacterial properties of the tested standard SiO_2_ fibres. Although the hybrid fibrous sample (DACHsilane) still shows a widespread attachment of both bacterial strains on its surface, it is important to note that most of them have a red fluorescence (Figure 6b,d), indicating a higher number of dead *S. aureus* and *P. aeruginosa* bacterial cells than viable (green) ones. Moreover, the bacterial adhesion to the surface of hybrid fibres is significantly weaker compared to the adhesion of the tested standard material for both bacterial strains. This observation is in agreement with the literature, where the antibacterial behaviour of similar molecule-based DACH derivatives was described [6]. The obtained results indicate that the activity of hybrid fibrous samples against *P. aeruginosa* is stronger in direct contact (Figure 6d).

### 3.3. Cytocompatibility Assessments

**Figure 7:**
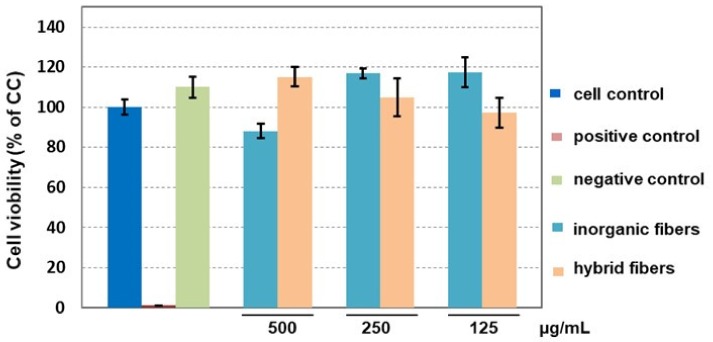
Comparison of the impact of hybrid DACHsilane nanofibres on the viability of 3T3-A31 cells compared to inorganic silica dioxide nanofibres as a control.

The cell viability assay performed on 3T3-A31 murine fibroblasts proved the cytocompatibility of the hybrid DACHsilane fibres in all three tested concentrations due to the viability highly exceeding the 70% CC level. As shown in Figure 7, exposure to the DACHsilane fibre extract led to increased cell viability in the case of the 500 µg/mL and 250 µg/mL extracts. The DACHsilane extracts had a concentration-dependent effect on cells as an increase in the extract concentration led to an increase in the cell viability from 97.2 ± 7.3% (125 µg/mL) up to 115.1 ± 4.9% (500 µg/mL). This led us to believe that the degradation products of the DACHsilane fibres are favouring cell proliferation. This effect was also observed with the inorganic silica dioxide nanofibres but in lower concentrations below 250 µg/mL (Figure 7). These findings were supported by a cell morphology analysis (see Figure S6 in the Supplement).

## 4. Conclusions

Homogeneous, purely organosilane fibres consisting of a (1*S*,2*S*)-cyclohexane-1,2-diamine bis-silane derivative in combination with TEOS were prepared for the first time in a one-pot sol-gel synthesis and electrospinning. The spinnable organosilane solution was acquired starting from a suitable choice of sol-gel processing parameters without the aid of electrospinning polymeric or surfactant easers. The resulting hybrid organosilane fibrous mat was confirmed to be uniform, thermally stable up to 200 °C, and sufficiently elastic. The prepared hybrid fibrous samples showed very promising antibacterial activity, particularly in direct contact with both of the tested pathogenic bacterial strains *S. aureus* and *P. aeruginosa*. These findings indicate that the organosilane fibres on the basis of (1*S*,2*S*)-cyclohexane-1,2-diamine may be a promising tool in the framework of the global issue of drug resistance caused by pathogenic bacteria. The assumptions made are also supported by the cytocompatibility study proving their biocompatibility and positive impact on fibroblasts viability, which may lead to a positive impact on wound healing.

## Figures and Tables

**Table 1:** Sols tested in electrospinning and conditions employed for the jet needle-electrospinning.

Sol-Gel Parameters		Parameters of Needle-Electrospinning				
Molar Ratio r = [H_2_O]/[silanes]	Molar Ratio Alc = [EtOH]/[silanes]	Viscosity [mPa·s]	Feeding Rate [mL·h^−1^]	Tip-To-Collector Distance [cm]	High Voltage [kV]	Temperature/Relative Humidity
2.0	9.7	40–60	0.5–1	15–20	20–25	25 °C/30%

**Table 2:** Composition of the siloxane fraction defined as the total amount of individual T*^n^* and Q*^n^* structure units in molar %.

Relative Amount of Building Units, %							RATIO Σ T*^n^*:Q*^n^*
**T^1^**	**T^2^**	**T^3^**	**Q^1^**	**Q^2^**	**Q^3^**	**Q^4^**	
1	27	38	0	11	20	3	66:34

## Supplementary Materials

The following are available online at https://www.mdpi.com/2073-4360/12/1/206/s1, Figure S1: Thermogravimetric analysis of the prepared hybrid DACHsilane fibers coupled with FTIR spectroscopy; Figure S2: Qualitative test describing the inhibition zones for standard sample—pure SiO_2_ fibers a) and hybrid DACHsilane fibers b) against *S. aureus*; pure SiO_2_ fibers c) and hybrid DACHsilane fibers d) against *P. aeruginosa*. Scale bar 1cm; Figure S3: Control related to the bacterial cell growth of *Staphylococcus aureus* (S.A.) and *Pseudomonas aeruginosa* (P.A.). Both bacterial strains were diluted 100 times. Scale bar 1 cm; Figure S4: The bacterial cell growth of *Staphylococcus aureus* (S.A.) on the inorganic fiber sample a) and on the hybrid fiber sample b). Scale bar 1 cm; Figure S5: The bacterial cell growth of *Pseudomonas aeruginosa* (P.A.) on the inorganic fiber sample a) and on the hybrid fiber sample b). Scale bar 1 cm; Figure S6: The 3T3-A31 cells spindle-like morphology after 24 hours exposure to the DACHsilane fibres extracts (a) 0 µg/mL (CC), (b) 125 µg/mL, (c) 250 µg/mL and (d) 500 µg/mL Merge of modular contrast vizualization and nucleus staining (DAPI) (Leica DMi8, obj. 20×). Scale bar 100 µm; Table S1: Antibacterial activity of the tested fibrous samples against *Staphylococcus aureus* and *Pseudomonas aeruginosa*.
