# Supplementary material for: (1S,2S)-Cyclohexane-1,2-diamine-based Organosilane Fibres as a Powerful Tool Against Pathogenic Bacteria
Source: Polymers (Basel). 2020 Jan 14;12(1):206. doi: 10.3390/polym12010206 (PMC7023662; doi:10.3390/polym12010206)
Supplement: Supplementary file 1 [file polymers-12-00206-s001.pdf]

polymers

MDPI

Supplementary

(1*S*,2*S*)-Cyclohexane-1,2-diamine-based organosilane fibres as a powerful tool against pathogenic bacteria

Veronika Máková[1,*], Barbora Holubová[1], David Tetour[2], Jiří Brus[3], Michal Řezanka[1], Miroslava Rysová[4] and Jana Hodačová[2]

[1] Department of Nanomaterials in Natural Science, Institute for Nanomaterials, Advanced Technologies and Innovation, Technical University of Liberec, Studentská 1402/2, 461 17 Liberec, Czech Republic
[2] Department of Organic Chemistry, University of Chemistry and Technology, Prague, Technická 5, 166 28 Prague, Czech Republic
[3] Institute of Macromolecular Chemistry, Academy of Sciences of the Czech Republic, Heyrovsky Sq. 2, 162 06 Prague, Czech Republic
[4] Department of Nanomaterials and Informatics, Institute for Nanomaterials, Advanced Technologies and Innovation, Technical University of Liberec, Studentská 1402/2, 461 17 Liberec, Czech Republic
* **Corresponding author:** veronika.makova@tul.cz; Phone: +420 485 353 863

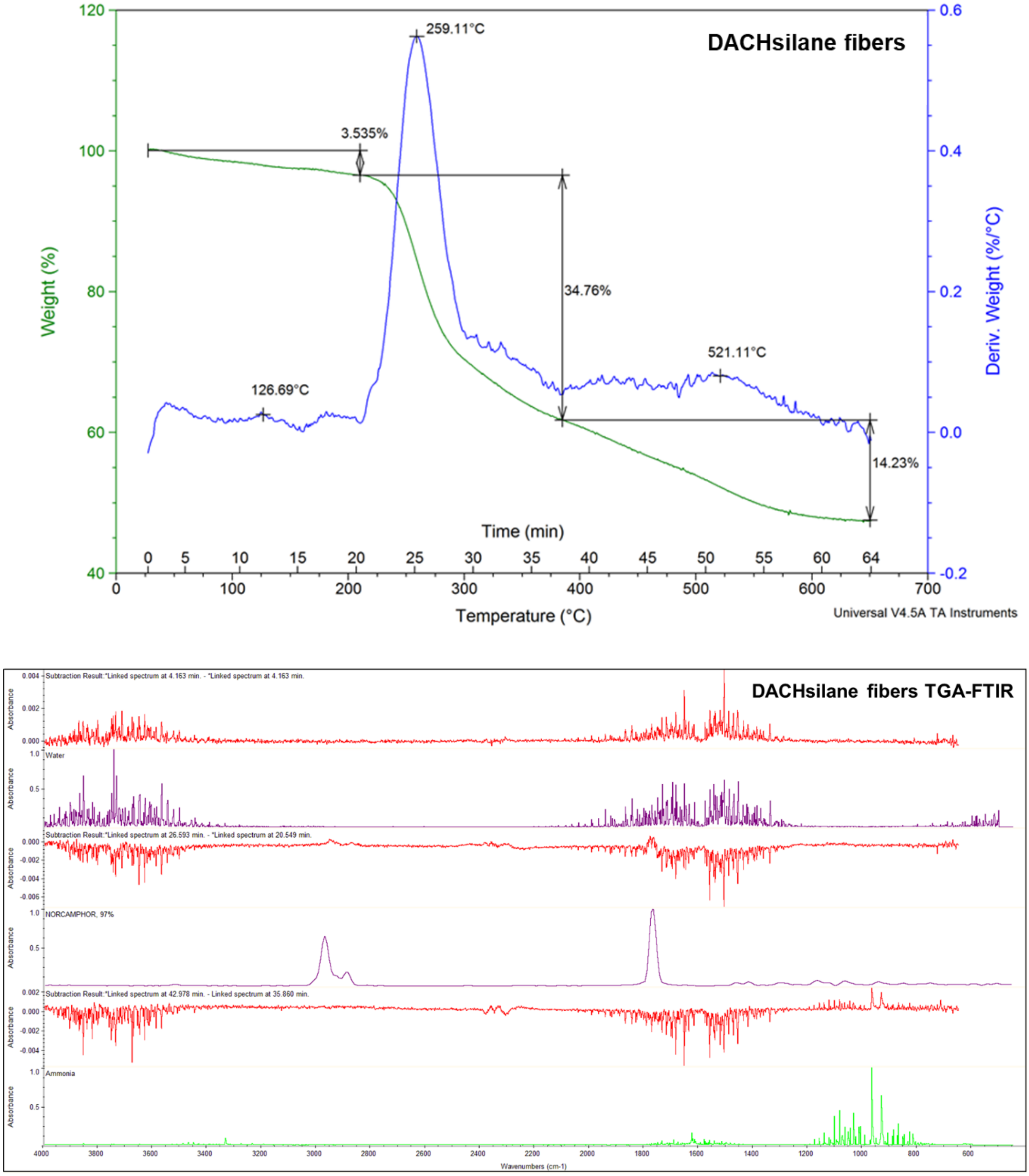

Figure S1. Thermogravimetric analysis of the prepared hybrid DACHsilane fibers coupled with FTIR spectroscopy.

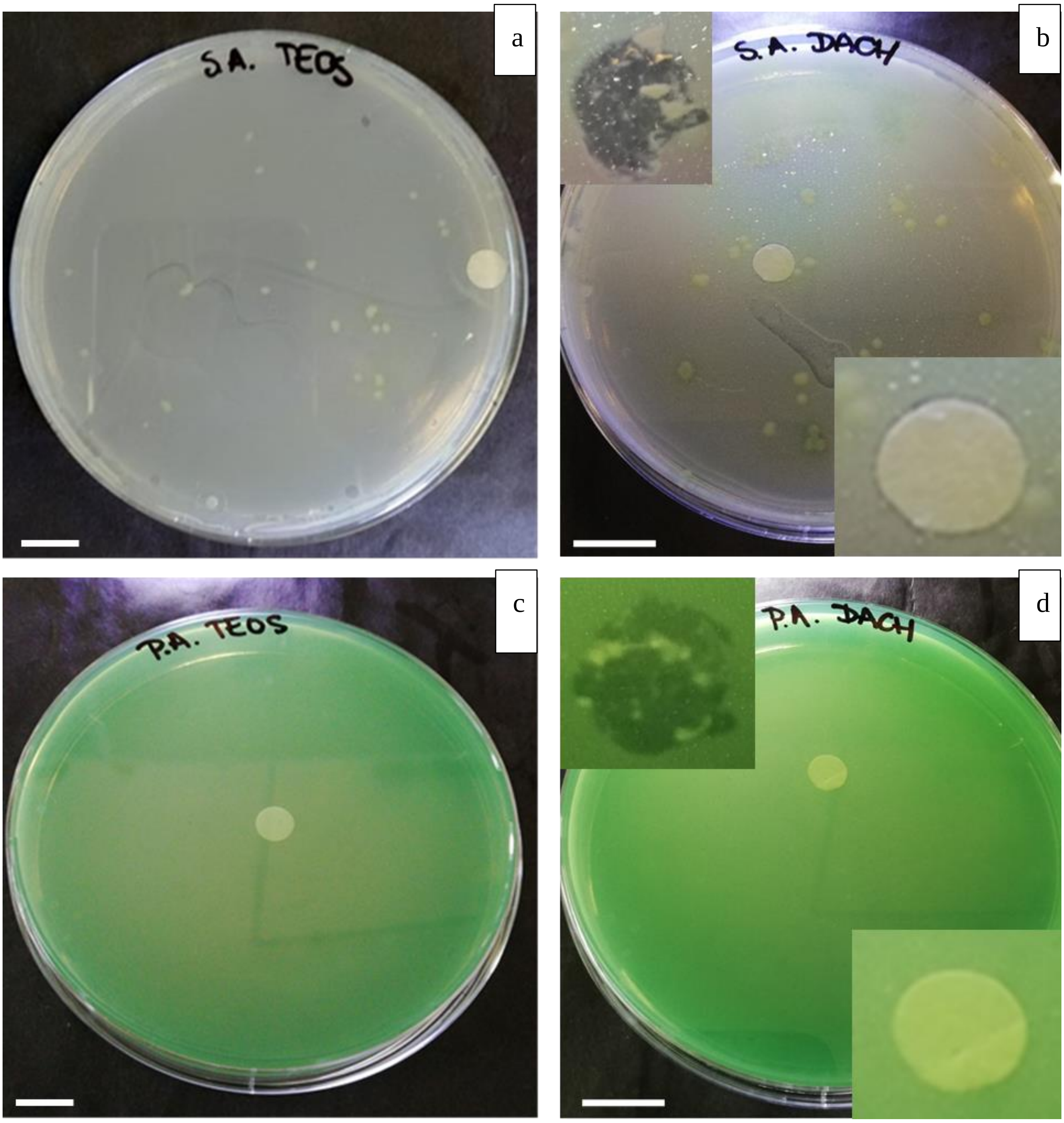

Figure S2. Qualitative test describing the inhibition zones for standard sample—pure SiO_2 fibers **a**) and hybrid DACHsilane fibers **b**) against *S. aureus*; pure SiO_2 fibers **c**) and hybrid DACHsilane fibers **d**) against *P. aeruginosa*. Scale bar 1cm.

The inhibition of bacteria for both strains was observed below the hybrid DACHsilane fibres (inset images **Figure S2b,d** on the left sides) Moreover, the DACHsilane fibres showed a halo zone hint around the sample in the case of *S. aureus* (**Figure S2b**—inset image on the right).

Table S1. Antibacterial activity of the tested fibrous samples against *Staphylococcus aureus* and *Pseudomonas aeruginosa*.

	**Staphylococcus aureus CFU/ mL**			**Pseudomonas aeruginosa CFU/ mL**		
	**Control**	**Inorganic fibers**	**Hybrid fibers**	**Control**	**Inorganic fibers**	**Hybrid fibers**
	>1000			>1000		
**1**		5	0		132	30
**2**		29	1		156	6
**3**		8	3		112	17

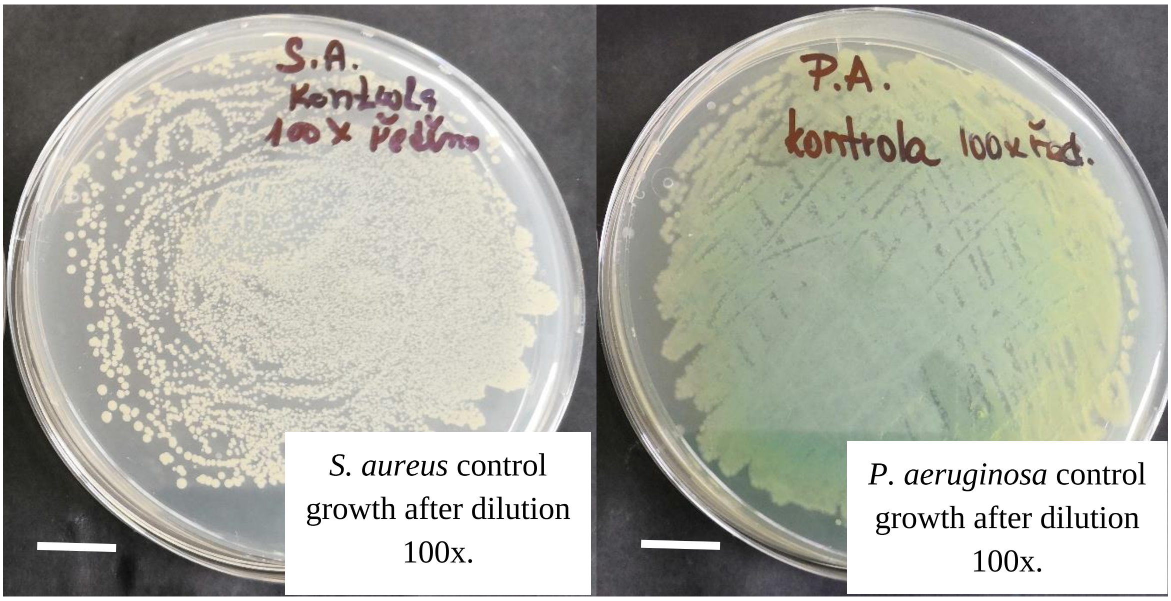

Figure S3. Control related to the bacterial cell growth of *Staphylococcus aureus* (S.A.) and *Pseudomonas aeruginosa* (P.A.). Both bacterial strains were diluted 100 times. Scale bar 1 cm.

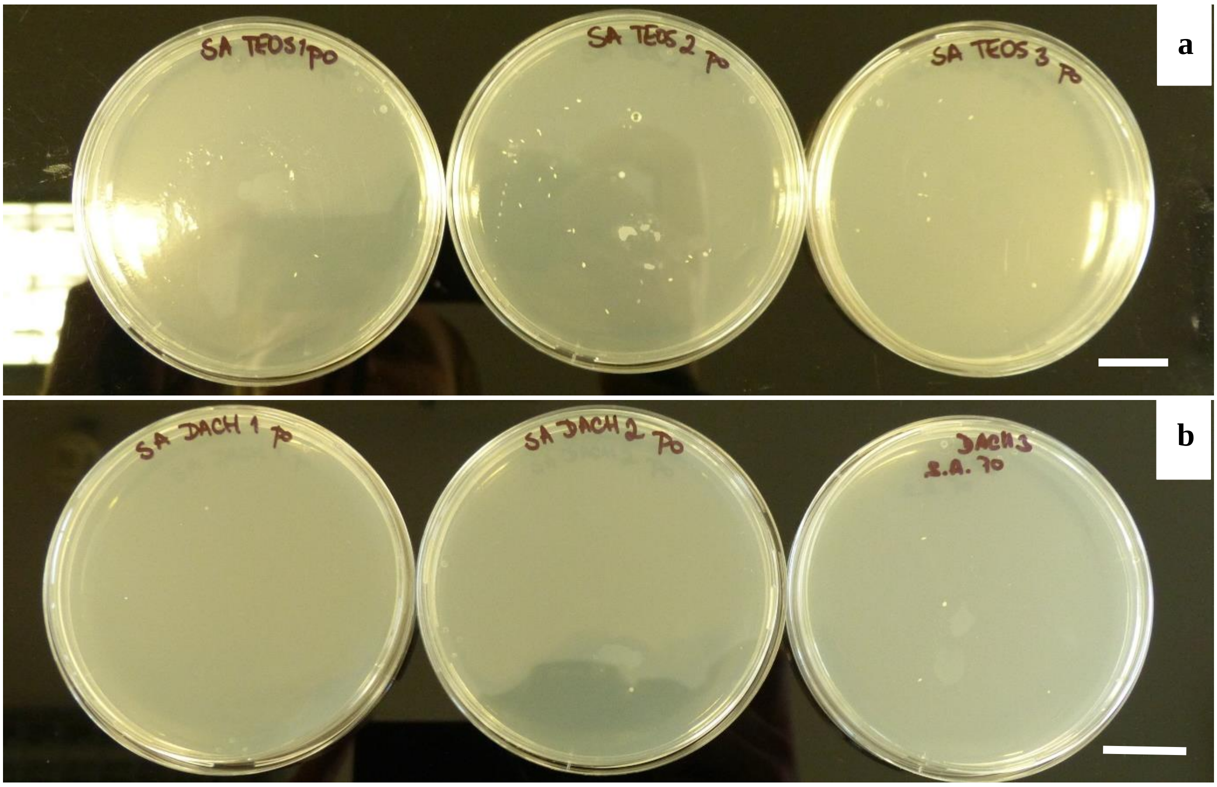

Figure S4. The bacterial cell growth of *Staphylococcus aureus* (S.A.) on the inorganic fiber sample **a**) and on the hybrid fiber sample **b**). Scale bar 1 cm.

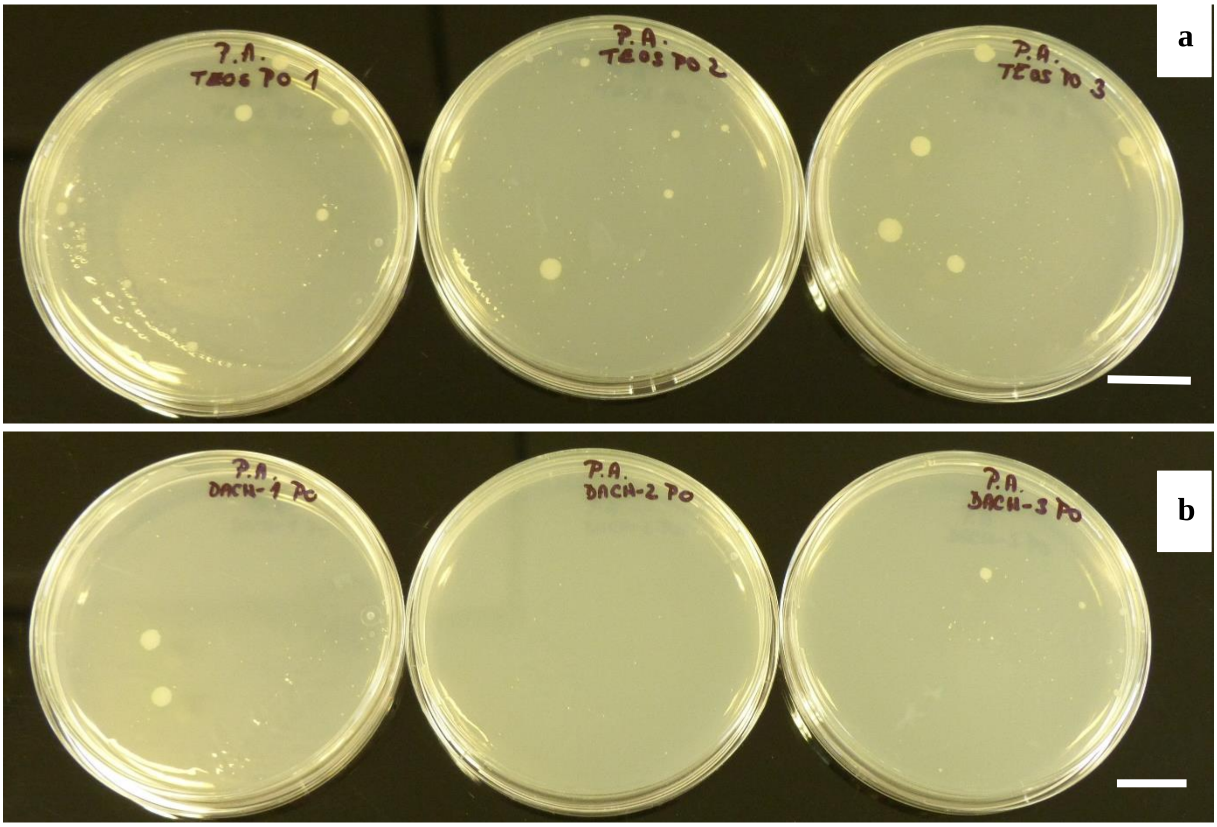

Figure S5. The bacterial cell growth of *Pseudomonas aeruginosa* (P.A.) on the inorganic fiber sample **a**) and on the hybrid fiber sample **b**). Scale bar 1 cm.

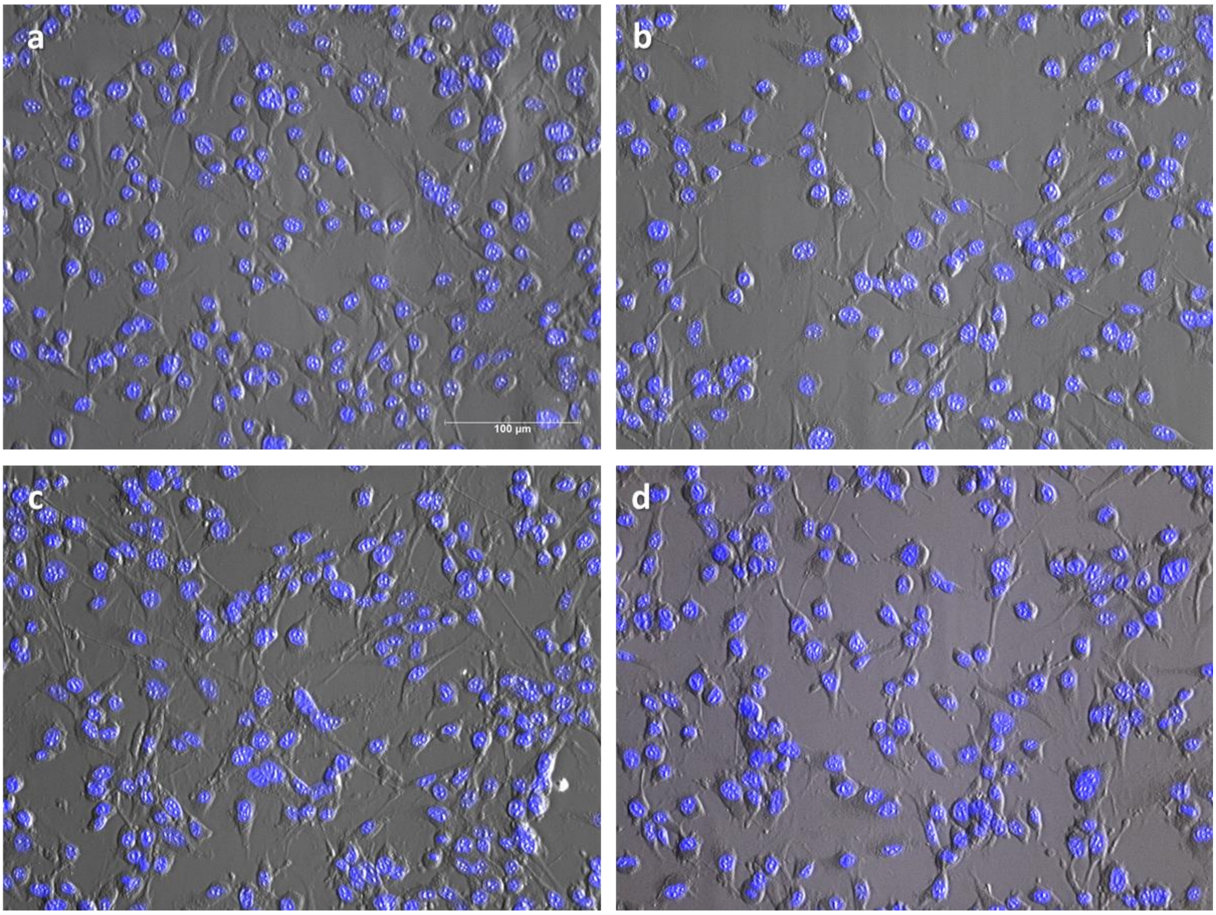

Figure S6. The 3T3-A31 cells spindle-like morphology after 24 hours exposure to the DACHsilane fibres extracts (**a**) 0 μg/mL (CC), (**b**) 125 μg/mL, (**c**) 250 μg/mL and (**d**) 500 μg/mL Merge of modular contrast vizualization and nucleus staining (DAPI) (Leica DMi8, obj. 20×). Scale bar 100 μm.

© 2020 by the authors. Submitted for possible open access publication under the terms and conditions of the Creative Commons Attribution (CC BY) license (http://creativecommons.org/licenses/by/4.0/).
